# A coordination polymer with unusual structural features from imidazolylbutyric acid and titanium isopropoxide

**DOI:** 10.1007/s00706-014-1245-2

**Published:** 2014-06-05

**Authors:** Matthias Czakler, Michael Puchberger, Christine Artner, Ulrich Schubert

**Affiliations:** Institute of Materials Chemistry, Vienna University of Technology, Vienna, Austria

**Keywords:** Titanium alkoxides, Carboxylate derivatives, Coordination polymers, Structure analysis

## Abstract

**Abstract:**

The coordination polymer [Ti(O*i*Pr)_3_(OOCCH_2_CH_2_CH_2_C_3_N_2_H_3_)]_*n*_ was prepared from 4-(imidazol-1-yl)butyric acid and titanium isopropoxide. The structure of the compound is remarkable, as the carboxylate group is coordinated in a chelating manner and no dimerization of the Ti(O*i*Pr)_3_ groups through OR bridges was observed.

**Graphical abstract:**

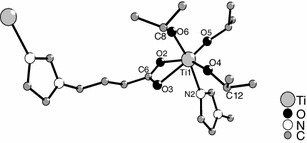

## Introduction

**Fig. 1:**
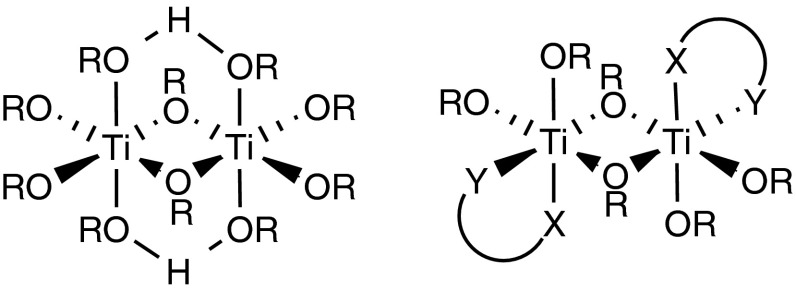
General structure of the adducts Ti(OR)_4_(LB) (*left*) and the mono-substituted derivatives Ti(OR)_3_(CL) (*right*, X∩Y = chelating ligand)

The chemistry of titanium alkoxide derivatives with organic co-ligands [1, 2] is of topical interest because of the importance of such compounds in sol–gel and CVD processes. Two main types of derivatives are known: (1) adducts of neutral Lewis bases (LB) of the general composition Ti(OR)_4_(LB), and (2) derivatives Ti(OR)_4−*x*_(CL)_*x*_ where one or more OR groups of Ti(OR)_4_ were substituted by an anionic chelating ligand (CL) and which are prepared by reaction of Ti(OR)_4_ with CL–H. The structures of mono-substituted derivatives Ti(OR)_3_(CL) with *β*-diketonates [3], aminoalcoholates [4, 5], *β*-aminocarboxylates [6], oximates [7, 8], and others as ligands are related to that of the adducts Ti(OR)_4_(LB) with alcohols [9–11] or amines as bases [12–16]. Both types are OR-bridged dimers with octahedrally coordinated titanium atoms. In the derivatives Ti(OR)_3_(CL), the neutral ligand and one neighboring OR group of Ti(OR)_4_(LB) are replaced by the chelating ligand CL (Fig. 1).

Reactions of Ti(OR)_4_ with carboxylic acids are special cases. In the first step, one OR group is substituted by a carboxylate ligand. The few derivatives which were isolated and structurally characterized have either the composition [Ti(OR)_3_(OOCR′)]_2_, with bridging carboxylate ligands [17], or [Ti(OR)_3_(OOCR′)(ROH)]_2_, where η_1_-carboxylate ligands are hydrogen-bonded to the coordinated alcohol at the neighboring titanium atom [17, 18]. In most reactions, however, carboxylate-substituted oxo/alkoxo clusters Ti_*a*_O_*b*_(OR)_*c*_(OOCR′)_*d*_ were obtained [1, 2]. This is due to ester formation between the alcohol cleaved in the first step and the employed carboxylic acid. The latter reaction produces water which is the source of the oxo ligands in the clusters.

In the light of the known structural chemistry of Ti(OR)_4_ derivatives, the outcome of the reaction of Ti(O*i*Pr)_4_ with 4-(imidazol-1-yl)butyric acid, which is reported in this article, is surprising, because the obtained coordination polymer has several unexpected features.

## Results and discussion

[Ti(O*i*Pr)_3_(OOCCH_2_CH_2_CH_2_C_3_N_2_H_3_)]_*n*_ (**1**) was obtained by reaction of Ti(O*i*Pr)_4_ with an equimolar amount of 4-(imidazol-1-yl)butyric acid (L–H) in isopropanol (Scheme 1).

**Figure:**
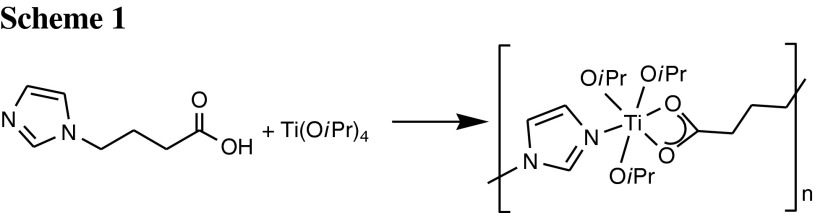

**Fig. 2:**
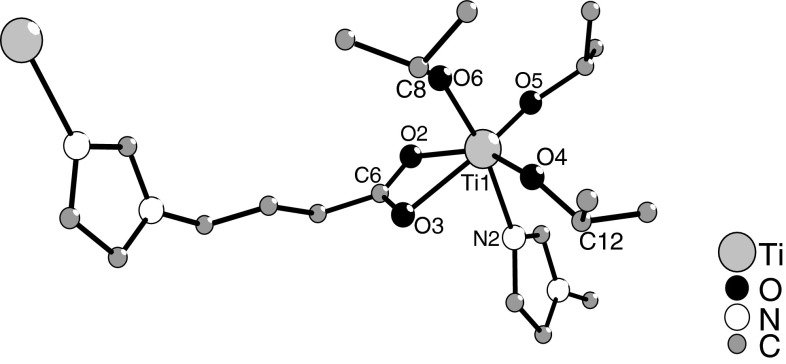
Ball and stick plot of **1**. Hydrogen atoms were omitted for clarity. Selected bond lengths/pm and angles/°: Ti(1)–O(1) 183.42(11), Ti(1)–O(2) 217.34(11), Ti(1)–O(3) 217.82(11), Ti(1)–O(4) 177.80(11), Ti(1)–O(5) 181.40(11), Ti(1)–N(2) 223.79(13); O(1)–Ti(1)–O(4) 98.03(5), O(1)–Ti(1)–O(5) 99.55(5), O(4)–Ti(1)–O(5) 108.2(5), N(2)–Ti(1)–O(2) 81.97(4), N(2)–Ti(1)–O(3) 80.56(4), O(1)–Ti(1)–N(2) 168.91(5)

**Fig. 3:**
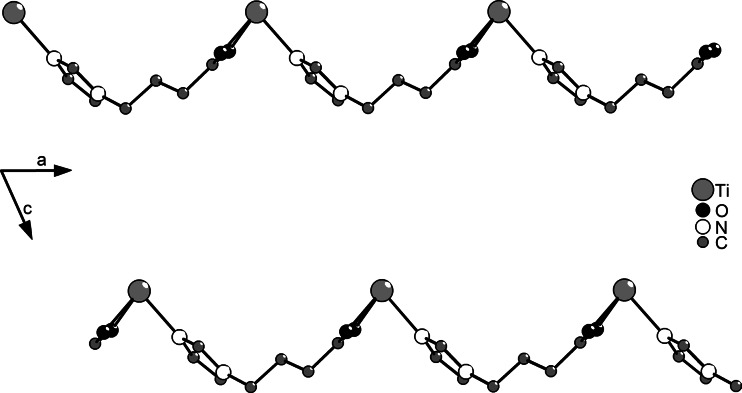
Packing of the chains perpendicular to the *b*-axis. Isopropoxo ligands and hydrogen atoms were omitted for clarity

In the crystalline state, compound **1** is a coordination polymer with mononuclear Ti(O*i*Pr)_3_ units as connector and L as linker, coordinating through the carboxylate group to one Ti atom and the imidazolyl group to the next (Fig. 2). Although the alkyl chain is highly flexible, no back-biting of one of the imidazolyl nitrogen atoms to the same titanium atom was observed. The polymer chain extends parallel to the *a*-axis (Fig. 3). The titanium atoms are six-coordinate with a distorted octahedral coordination geometry and the O*i*Pr ligands in a *mer* arrangement. Distortion of the polyhedron is due to the chelating carboxylate group and results in O–Ti–O bond angles of the O*i*Pr ligands between 99.55°  and 108.2°. The coordinating nitrogen atom is slightly tilted towards the carboxylate ligand. The Ti(1)–N(2) distance is distinctly shorter than that observed in the Ti(OR)_4_(amine) adducts with primary amines, which are typically in the range 129–131 pm, or in Ti(OR)_*x*_ derivatives with DHP ligands (226 pm, DHP-H_2_ = 4,6-dihydroxypyrimidine) [19].

**Fig. 4:**
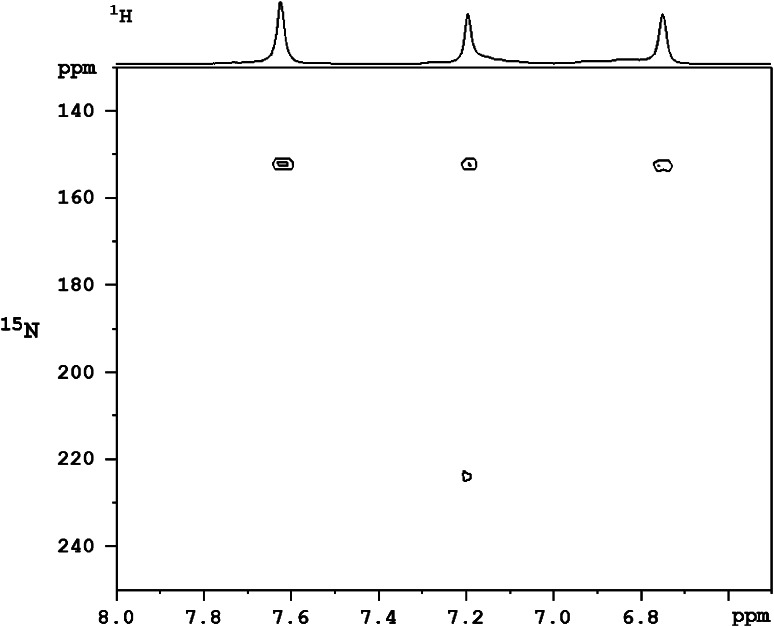
^1^H/^15^N HMBC NMR spectrum of the complex **1** in CD_2_Cl_2_

The solution ^1^H NMR spectrum of **1** shows one doublet at 1.22 ppm for the terminal CH_3_ of the O*i*Pr ligands. The signals of the imidazolyl group appear at 6.73, 7.19, and 7.59 ppm. To verify the coordination behavior of the imidazolyl group in solution, 2D correlation spectra (^1^H/^15^N HMBC) of L–H and the complex were recorded. The spectrum of the complex (Fig. 4) shows correlations at 152 and 224 ppm, while the spectrum of the free ligand shows correlations at 153 and 239 ppm. The shift difference of 15 ppm for one of the two nitrogen atoms is an indication that coordination is retained in solution.

## Conclusions

Reaction of Ti(O*i*Pr)_4_ with 4-(imidazol-1-yl)butyric acid resulted in the formation of the coordination polymer [Ti(O*i*Pr)_3_(OOCCH_2_CH_2_CH_2_C_3_N_2_H_3_)]_*n*_ (**1**). Only few one-dimensional titanium-containing coordination polymers were hitherto structurally characterized [20], among them also adducts of Ti(OR)_4_ with diamines [14–16]. A metal–organic framework with Ti_8_O_8_(OH)_4_ units as connector and terephthalate linkers was obtained from Ti(O*i*Pr)_4_ and terephthalic acid [21], and materials at the borderline between sol–gel and metal–organic framework structures in the reaction with tri- and tetracarboxylic acids [22]. On the other hand, amino-substituted carboxylic acids with rigid structures often give rise to the formation of coordination polymers [23].

Apart from the fact that a coordination polymer with Ti(O*i*Pr)_3_ units was formed, compound **1** shows several remarkable structural features. First, to the best of our knowledge, this is the first titanium alkoxide derivative with a chelating carboxylate group (in all other examples, the carboxylate group bridges two titanium atoms). Second, the fact that coordination of the imidazoyl group inhibits dimerization through alkoxo bridges is quite unusual, because completion of the octahedral coordination sphere of titanium through OR bridges is expected to be more favorable than coordination of a neutral nitrogen donor. A related example is [Ti(OCH_2_CMe_3_)_3_(py)]_2_(μ-DHP) with very bulky OR ligands [19]. Third, no oxo cluster is formed. The formation of the coordination polymer apparently inhibits ester formation. This was already observed when Ti(O*i*Pr)_4_ was reacted with di-, tri- and tetracarboxylic acids [21, 22].

## Experimental

All operations were carried out in a moisture- and oxygen-free argon atmosphere using Schlenk techniques. Isopropanol was dried by refluxing twice over sodium metal and distillation. The solvents for NMR spectroscopy (Eurisotop) were degassed prior to use and stored over molecular sieve. ^1^H and ^13^C solution NMR spectra were recorded on a Bruker AVANCE 250 (250.13 MHz {^1^H}, 62.86 MHz {^13^C}). Correlation spectra were recorded on a Bruker AVANCE DPX 300 (300.13 MHz {^1^H}, 30.42 MHz {^15^N}). Both spectrometers were equipped with a 5-mm inverse-broadband probe head and a *z*-gradient unit. 2D experiments were measured with Bruker standard pulse sequences: HMBC (Heteronuclear Multiple Bond Correlation). ^1^H/^15^N HMBC spectra of **1** and L were recorded in CD_2_Cl_2_ and DMSO, respectively.

### *Titanium tris(isopropoxo) 4*-*(imidazol*-*1*-*yl)butanate* (**1**)

Ti(O*i*Pr)_4_ was obtained from ABCR and used as received. Ti(O*i*Pr)_4_ (1.1 cm^3^, 3.8 mmol) was added to a solution of 590 mg of 4-(imidazol-1-yl)butyric acid (3.8 mmol) [24] in 3 cm^3^ of water-free isopropyl alcohol under argon. Crystals of **1** were obtained after 4 weeks (yield 450 mg, 31 %). ^1^H NMR (CDCl_3_, 250 MHz): *δ* = 1.21 (d, 18H, CH_3_), 1.83 (2H, CH_2_), 2.05 (2H, CH_2_), 3.78 (2H, CH_2_), 4.70 (3H, CH_3_), 6.74 (1H, CH), 7.17 (1H, CH), 7.59 (1H, CH) ppm; ^13^C NMR (CDCl_3_, 62.90 MHz): *δ* = 25.96 (CH_3_), 26.69 (CH_2_), 32.64 (CH_2_), 46.67 (CH_2_), 77.36 (CHMe_2_), 118.29 (CH), 129.75 (CH), 138.07 (CH), 186.50 (COO) ppm; IR (ATR): $\bar{\nu }$ = 3,144 (vw), 2,963 (w), 2,927 (vw), 2,860 (vw), 1,724 (vvw), 1,591 (m), 1,540 (m), 1,520 (w), 1,462 (m), 1,443 (w), 1,373 (w), 1,359 (w), 1,327 (w), 1,226 (w), 1,161 (w), 1,118 (s), 1,084 (m), 1,020 (w), 980 (m), 942 (w), 845 (w), 764 (w), 733 (m), 665 (w) cm^−1^; IR (CHCl_3_): $\bar{\nu }$ = 2,971 (s), 2,930 (m), 2,864 (w), 1,639 (m), 1,570 (s), 1,513 (w), 1,441 (m), 1,376 (s), 1,283 (w), 1,126 (s), 1,071 (m), 1,042 (w), 1,004 (m), 946 (m), 911 (w), 888 (w), 853 (w) cm^−1^.

### X-ray structure analysis

All measurements were performed using MoK_α_ radiation (*λ* = 71.073 pm). Data were collected on a Bruker AXS SMART APEX II four-circle diffractometer with κ-geometry at 100 K with φ and ω-scans and 0.5° frame width (Table 1). The data were corrected for polarization and Lorentz effects, and an empirical absorption correction (SADABS) was applied. The cell dimensions were refined with all unique reflections. SAINT PLUS software (Bruker Analytical X-ray Instruments, 2007) was used to integrate the frames. Symmetry was checked with the program PLATON.

**Table 1:** Crystal data and structure refinement details for **1**

Empirical formula	C_16_H_30_N_2_O_5_Ti
Formula weight	378.32
Crystal system	Triclinic
Space group	$P_{{\bar{1}}}$
*a*/pm	956.79(6)
*b*/pm	1072.38(6)
*c*/pm	1121.04(7)
*α*/°	85.272(2)
*β*/°	65.808(3)
*γ*/°	68.423(2)
*V*/pm^3^ × 10^6^	972.53(10)
*Z*	2
*D*_*x*_/Mg m^−3^	1.292
*µ*/mm^−1^	0.466
Crystal size/mm^3^	0.2 × 0.14 × 0.12
No. meas. refl.	20,370
Obs. refl. [*I* > 2*σ* (*I*)]	4,207
*R*_int_	0.0359
*θ* max/°	29.62
*R* [*F* ^2^ > 2*σ*(*F*)], w*R* (*F* ^2^), *S*	0.0374, 0.0915, 1.050
No. reflections/parameters	5298/253
Weighting scheme	*w* = 1/[*σ* ^2^(*Fo* ^2^) + (0.0387*P*)^2^ + 0.3848*P*]
*δρ*_max_, *δρ*_min_/e 10^−6^ pm^−3^	0.501, −0.481

The structure was solved by the Patterson method (SHELXS97). Refinement was performed by the full-matrix least-squares method based on *F* ^2^ (SHELXL97) with anisotropic thermal parameters for all non-hydrogen atoms. Hydrogen atoms were inserted in calculated positions and refined riding with the corresponding atom. The carbon atoms of one O*i*Pr ligand of **1** were disordered. Their two positions were refined with about 40 and 60 % occupancy.

CCDC-965819 contains the supplementary crystallographic data for **1**. These data can be obtained free of charge from The Cambridge Crystallographic Data Centre via www.ccdc.cam.ac.uk/data_request/cif.
